# Management of chronic anal fissures: should fissurectomy be performed with botulinum toxin injection?

**DOI:** 10.1007/s10151-025-03153-z

**Published:** 2025-05-22

**Authors:** R. Quinn, J. Ellis-Clark, S. Albayati

**Affiliations:** 1 Department of Colorectal Surgery, Nepean Hospital, Derby St, Kingswood, NSW 2747 Australia; 2 Sydney Medical School, Faculty of Medicine and Health, University of Sydney, Sydney, NSW Australia

**Keywords:** Botulinum toxins, Fissure in ano, Treatment outcome, Fecal incontinence

## Abstract

**Background:**

Despite the superior success rate of lateral internal sphincterotomy for managing chronic anal fissure (CAF), there is a trend towards sphincter-preserving treatment due to the increased risk of incontinence. Botulinum toxin (BT) and fissurectomy are two sphincter-preserving options for CAF. We aim to assess if combining fissurectomy with botox treatment is superior to botulinum toxin alone in the management of CAF.

**Methods:**

This retrospective cohort study was conducted across two Sydney hospitals over 7 years. All patients with a CAF managed with either BT and fissurectomy (group 1) or BT only (group 2) were included. Primary outcome was healing rate defined as resolution or significant improvement of perianal symptoms at initial follow-up. Secondary outcomes were persistence, recurrence, re-intervention and faecal incontinence rate. Follow-up questionnaire was conducted to compare long-term outcomes between the two groups.

**Results:**

Fifty-seven patients met the inclusion criteria (group 1, 37; group 2, 20). Mean BT dose and injection location between the groups were similar (*p* = 0.259 and *p* = 0.427). There was a 65% response rate to the follow-up questionnaire. Median follow-up was 34.3 months (range 0.4–93). There was no difference in healing (56.7% vs. 50%, *p* = 0.561), recurrence (37.8% vs. 30%, *p* = 0.383) or re-intervention rate (13.5% vs. 20%, *p* = 0.888). Long-term incontinence rate was significantly higher in patients group 2 (0% vs. 10%, *p* = 0.010), with two patients reporting persistent flatus incontinence. Median overall satisfaction score was 3/4 (range 1–4), in both groups (*p* = 0.469).

**Conclusion:**

Botulinum toxin with or without fissurectomy is a safe sphincter-sparing treatment option for CAF. However, the addition of fissurectomy to BT does not improve healing rates and we therefore recommend BT injection alone as a second-line treatment of CAF in patients who fail topical treatment.

## Background

Anal fissure is defined as a small longitudinal tear in the squamous epithelium of the anal canal, commonly at the posterior midline position, resulting in debilitating pain [1]. Chronic anal fissures (CAF) persist for more than 6 weeks as a result of the increased tone in the internal anal sphincter (IAS), which leads to localised ischaemia and prevents healing. CAF are often associated with a hypertrophied anal papilla, sentinel tag and exposed IAS at the base [2–4].

Management of CAF targets the disease process, addressing the IAS spasm and the chronically fibrosed wound. Topical antispasmodic treatments with glyceryl trinitrate (GTN) and/or diltiazem have 52.3–63.8% success rates [3]. However, they are limited by their side-effect profile and require a long treatment course, which can lead to poor compliance [2, 5]. Failure of topical treatment is often followed by chemical sphincterotomy with botulinum toxin A (BT), which carries with it a risk of faecal incontinence, though usually transient [3–6]. Persistent fissures can be managed surgically with fissurectomy (FIS), anal advancement flap or lateral internal sphincterotomy (LIS). FIS promotes healing of the fissure by excising the chronic fibrotic edges and sentinel tag [7]. LIS has the highest success rates (> 95%) and lowest recurrence rates; however, LIS also has a reported incontinence rate of 3.9–10% in recent systematic reviews [3, 5, 8–11].

Despite the risk of long-term incontinence reported with LIS, the Association of Coloproctology of Great British and Ireland (ACPGBI) and the American Society of Colon and Rectal Surgeons (ASCRS) clinical practice guidelines recommend LIS should be considered for first-line treatment in appropriately selected and informed patients [2, 7]. However, international surveys of clinical practice by general and colorectal surgeons’ report that LIS is commonly reserved for refractory anal fissures [12–14]. Chemical sphincterotomy with BT has been extensively studied, and despite the lower healing rate and higher recurrence rate, it is the preferred management for CAF after topical measures. The addition of FIS is thought to promote healing by wound debridement, whilst the BT reduces the IAS spasm [15]. There are several cohort studies assessing the effectiveness of fissurectomy and BT on CAF, with only three small cohort studies directly comparing to BT as a single therapy [16–18]. We aim to assess if combining fissurectomy with BT treatment is superior to botulinum toxin alone in the management of CAF.

## Methods

This retrospective cohort study was conducted across two hospitals in Sydney, Australia, between January 2017 and December 2023. This study was approved by the Nepean Blue Mountains Local Health District Human Research Ethics Committee on 11 April 2024 in accordance with the National Health and Medical Research Council Act 1992 and the National Statement on Ethical Conduct in Human Research 2023 (ETH00431).

### Eligibility criteria

All adult patients diagnosed with a CAF, defined as an anal fissure that has persisted for more than 6 weeks and managed with botulinum toxin or botulinum toxin and fissurectomy were included. Exclusion criteria was as follows: (a) children (< 18 years); (b) acute anal fissure (duration < 6 weeks); (c) atypical fissure defined as those with a history of inflammatory bowel disease, immunocompromised patients, history of anal cancer or concurrent perianal fistulas or abscess.

### Procedure

All patients were managed under general anaesthetic in lithotomy or left lateral position treated by one of five colorectal surgeons. On the basis of findings and usual practice, the treating surgeon performed one of two procedures: (group 1) botulinum toxin injection and fissurectomy or (group 2) botulinum toxin injection only. Fissurectomy involved excision of the fibrosed edges with diathermy or scalpel, and debridement of the base of the fissure with a Raytec. The sentinel skin tag was excised, if present. Botulinum toxin A (BOTOX, AbbVie, Mascot, Australia) was injected as per the surgeon’s routine practice. This consisted of a dose of 25–100 IU and injection into either the intersphincteric space (ISS) or the IAS. Patients were discharged home the same day with outpatient follow-up.

### Outcomes measured

The primary outcome of this study was healing rate, defined as resolution or significant improvement of perianal symptoms at initial follow-up and not requiring any further surgical management. Secondary outcomes examined included (1) persistence rate, defined as no change or worsening of symptoms at first follow-up; (2) recurrence rate, defined as new symptoms after healing; (3) re-intervention rate, defined as further BT or surgical procedure; (4) faecal incontinence rate, assessed using the Pescatori grading system [19]; and (5) overall satisfaction score (1 = unsatisfied, 2 = neutral, 3 = satisfied, 4 = very satisfied).

### Data collection and analysis

Patients were identified through operation theatre lists at both hospital sites using the following keywords: ‘anal fissure’, ‘fissure’, ‘botox’, ‘botulinum toxin’ and/or ‘fissurectomy’. Data was collected on patient characteristics, operative findings and procedure performed, postoperative outcomes, complications and subsequent surgical procedures. Patients who met inclusion criteria were contacted by phone to complete a follow-up survey. Information on treatment satisfaction, current symptoms, treatment since last review and incontinence was collected.

Statistical analysis was conducted with IBM SPSS Statistics v29.0.1.0. Means, median, standard deviation (SD) and range were calculated for continuous data. Independent* t* test was conducted between the two groups for continuous data and chi-square test for categorical data. A Kaplan–Meier curve was calculated for rate of escalation to lateral sphincterotomy for management of persistent or recurrent fissure. All analyses will be considered significant if the* p* value is < 0.05.

## Results

A total of 57 patients were identified that met the inclusion criteria between January 2017 and December 2023. Thirty-seven patients were managed with BT and FIS (group 1), and the remaining 20 patients received BT only (group 2). The mean age was 40 ± 14.2 years (range 18–74 years). There was a female predominance in the BT and FIS group, although this was not significant (*p* = 0.083). The majority of patients reported pain (92.9%) and bleeding (52.6%) on presentation, with 21.1% of patients reporting a history of constipation. Significantly more patients failed topical treatment with either GTN or diltiazem in group 1 compared to group 2 (*p* = 0.005), whereas significantly more patients received a previous dose of BT in group 2 (3 vs. 1, *p* = 0.029). Patient demographics are presented in Table 1.

**Table 1:** Patient demographics

	Total (*n* = 57)	BT + FIS (*n* = 37)	BT (*n* = 20)	*p* value
Age, years (mean ± SD)	40 ± 14.2	39 ± 11.7 Range 22–66	45 ± 17.5 Range 18–74	0.076
Gender (M/F)	20:37	10:27	10:10	0.083
Symptoms, *n* (%)				
Pain	53 (92.9%)	36 (97.3%)	17 (85%)	0.083
Bleeding	30 (52.6%)	24 (64.8%)	6 (30%)	0.012
Constipation	12 (21.1%)	8 (21.6%)	4 (20%)	0.886
Lump	2 (3.5%)	1 (2.7%)	1 (5%)	0.653
Previous treatment, *n* (%)				
Laxatives	48 (84.2%)	34 (91.9%)	14 (70%)	0.523
GTN/diltiazem	36 (63.1%)	30 (81.1%)	6 (30%)	0.005
BT	4 (7%)	1 (2.7%)	3 (15%)	0.029
Dilatation	1 (1.7%)	0 (0%)	1 (5%)	0.118
Fissurectomy	2 (3.5%)	1 (2.7%)	1 (5%)	0.479

*BT* botulinum toxin, *FIS* fissurectomy, *GTN* glyceryl trinitrate ointment

The majority of fissures were posterior (68.4%); however, the ratio of fissure location was significantly different between the two groups (*p* = 0.038). There was no difference in mean BT dose (56.2 vs. 59.5, *p* = 0.259) with a range of 25–100 IU given. The majority of BT was injected into the ISS (70.2%), with two injections at the 3 and 9 o’clock position (75.4%), with no difference between the groups (*p* = 0.427 and *p* = 0.384, respectively). Procedure details for both groups are presented in Table 2.

**Table 2:** Procedure details

	Total (*n* = 57)	BT + FIS (*n* = 37)	BT (*n* = 20)	*p* value
Fissure location				
Anterior (12 o’clock)	13 (22.8%)	12 (32.4%)	1 (5%)	0.038
Posterior (6 o’clock)	39 (68.4%)	24 (64.8%)	15 (75%)	
Missing data	5 (8.7%)	1 (2.7%)	4 (20%)	
BT dose, IU (mean ± SD)	57.3 ± 17.3	56.2 ± 15.6 Range 25–100	59.5 ± 20.8 Range 30–100	0.259
Location of injection				
ISS	40 (70.2%)	27 (72.9%)	13 (65%)	0.427
IAS	16 (28.1%)	9 (24.3%)	7 (35%)	
Missing data	1 (1.7%)	1 (2.7%)	0 (0%)	
Injection sites				
3 and 9 o’clock	43 (75.4%)	29 (78.4%)	14 (70%)	0.384
Four quadrants	1 (1.7%)	0 (0%)	1 (5%)	
Circumferential	12 (21%)	8 (21.6%)	4 (20%)	
Missing data	1 (1.7%)	0 (0%)	1 (5%)	
Concurrent haemorrhoid procedure				
Sclerotherapy injection	6 (10.5%)	4 (10.8%)	2 (10%)	0.892
Artery ligation	4 (7%)	2 (5.4%)	2 (10%)	
Excision	2 (3.5%)	1 (2.7%)	1 (5%)	

*BT* botulinum toxin, *FIS* fissurectomy, *IU* international units, *ISS* intersphincteric space, *IAS* internal anal sphincter

### Healing

**Fig. 1:**
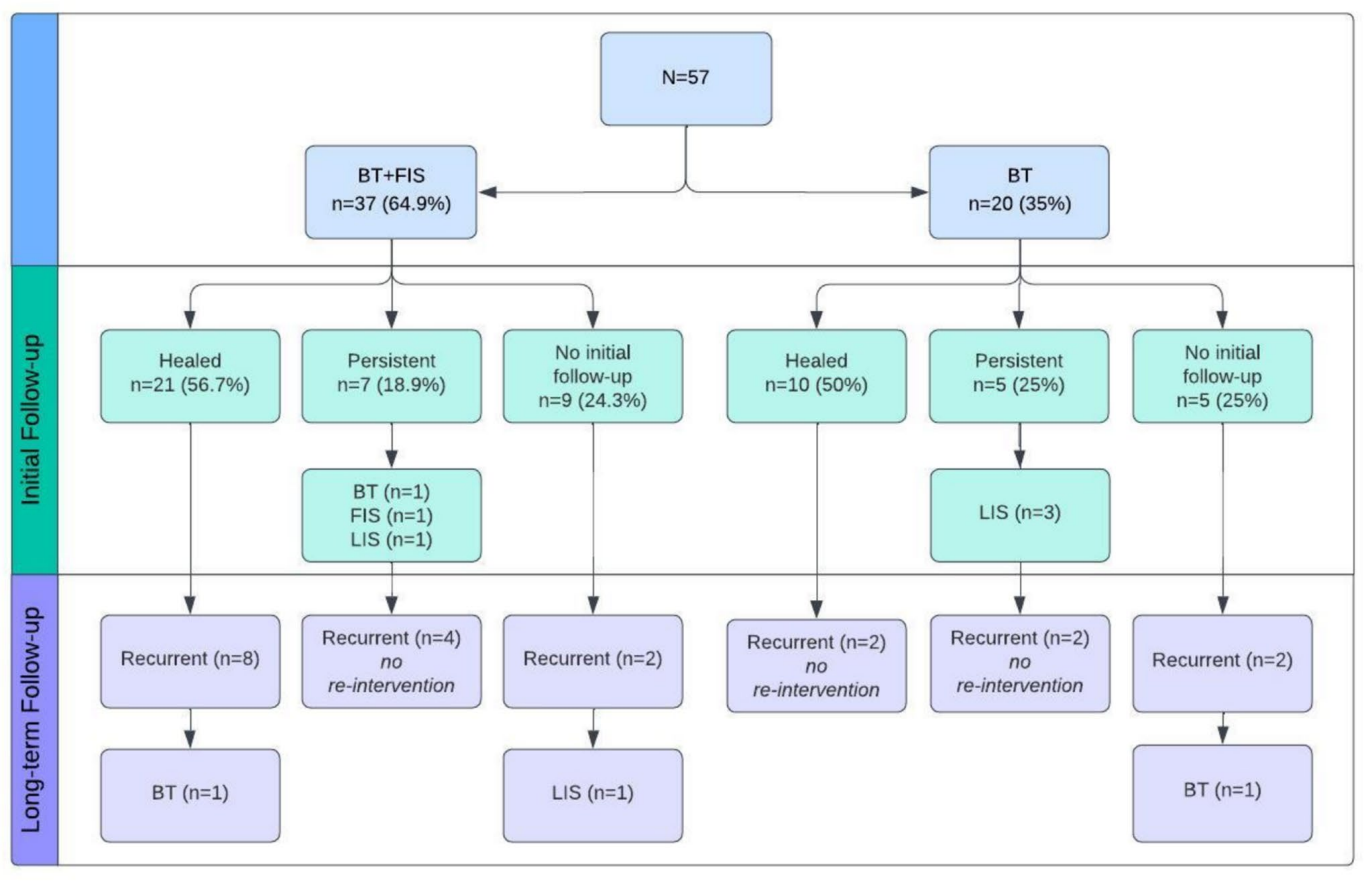
Flow diagram showing outcomes of patients after fissure management

The healing rate was 54.4%, with no significant difference between the two groups (56.7% vs. 50% respectively, *p* = 0.561). Initial follow-up was similar between the groups with a median 5.9 weeks (range 1.1–15.9 weeks, *p* = 0.127). However, 9 (24.3%) group 1 and 5 (25%) group 2 patients failed to follow-up after the procedure, resulting in missing data of initial outcomes. Patient outcomes are presented in the flowchart in Fig. 1 and Table 3.

**Table 3:** Short- and long-term outcomes

	Total (*n* = 57)	BT + FIS (*n* = 37)	BT (*n* = 20)	*p* value
Short-term				
First follow-up, weeks median (range)	5.9 (1.1–15.9)	5.4 (1.1–9.9)	6.0 (1.9–15.9)	0.127
Healed	31 (54.4%)	21 (56.7%)	10 (50%)	0.561
Persistence	12 (21%)	7 (18.9%)	5 (25%)	0.561
Re-intervention	6 (50%)	3 (42.8%)	3 (60%)	0.558
Faecal incontinence	5 (8.7%)	3 (8.1%)	2 (10%)	0.798
Long-term				
Long-term follow-up, months median (range)	34.3 (0.4–93)	37.1 (0.4–88.8)	11.5 (1.4–93.0)	0.156
Recurrence	20 (35.1%)	14 (37.8%)	6 (30%)	0.383
Re-intervention (overall)				
BT	3 (5.2%)	2 (5.4%)	1 (5%)	0.888
FIS	1 (1.7%)	1 (2.7%)	0 (0%)	
LIS	5 (8.7%)	2 (5.4%)	3 (15%)	
Long-term faecal incontinence				
Pescatori score (0–6)	0.2 ± 0.8	0.0 ± 0.0	0.8 ± 1.6	0.087
*n* (%)	2 (10%)	0 (0%)	2 (10%)	0.010
Satisfaction score (1–4) median (range)	3 (1–4)	3 (1–4)	4 (1–4)	0.469

*BT* botulinum toxin, *FIS* fissurectomy, *LIS* lateral internal sphincterotomy

### Persistence

In group 1, there were 7 (18.9%) patients with persistent symptoms, with only 3 (42.8%) requiring further surgical intervention; this included a repeat dose of BT, FIS alone and LIS. Similarly, in group 2, with a persistent rate of 25%, 3 (60%) patients required re-intervention, all undergoing a LIS. There was no significant difference in the rate of re-intervention for persistence symptoms between the groups (*p* = 0.558). Persistence was successfully managed with topical treatments and laxatives in the remaining patients.

### Recurrence and re-intervention

**Fig. 2:**
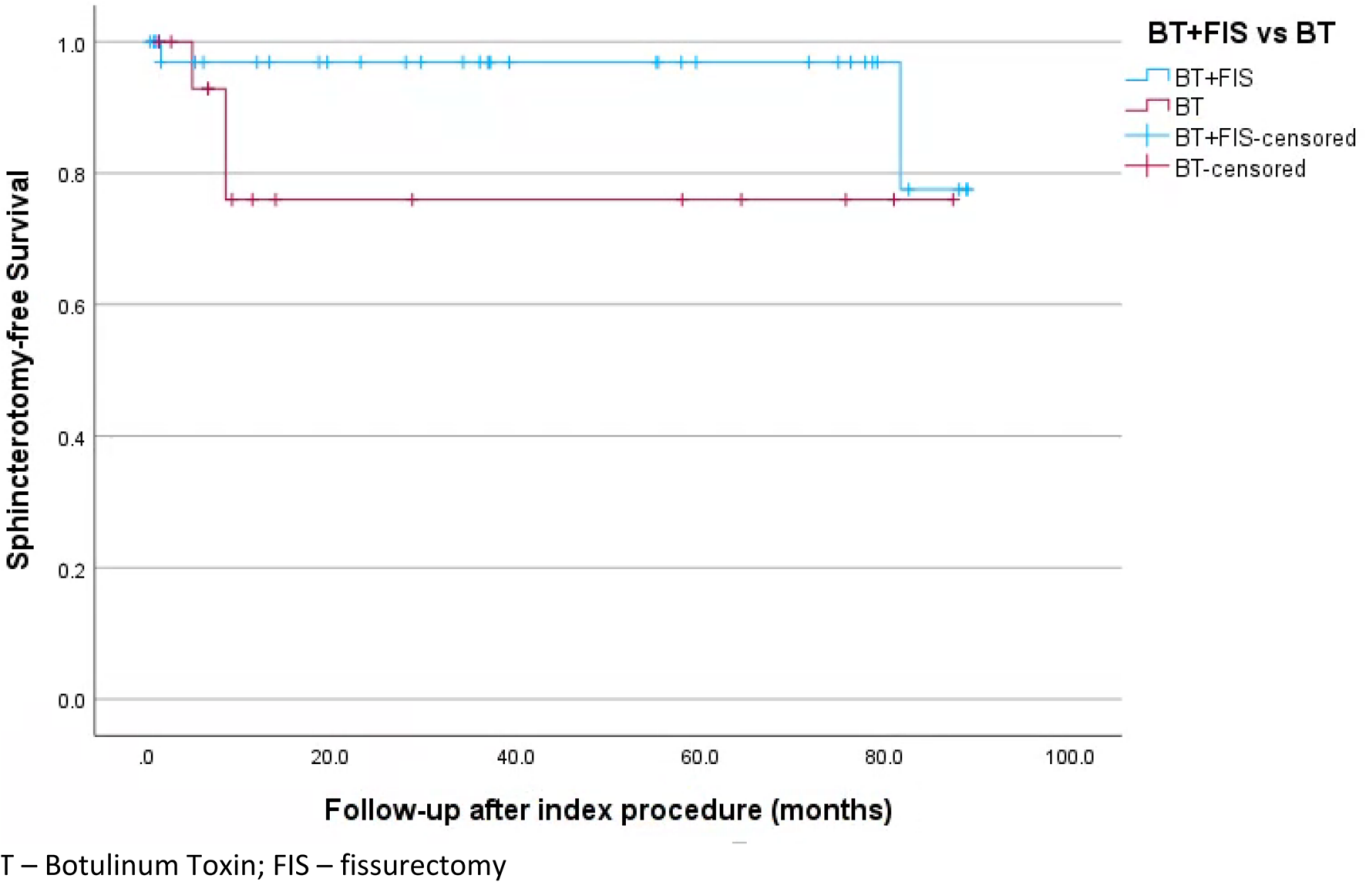
Rate of escalation to lateral sphincterotomy following failed sphincter-sparing treatment. *BT* botulinum toxin, *FIS* fissurectomy

Recurrence rate was similar between the two groups (37.8% group 1 vs. 30% group 2, *p* = 0.383), with only three patients requiring further surgical intervention. One patient who required a LIS after persistent symptoms following initial BT treatment (group 2) had further recurrence managed with topical ointments. All other patients who underwent LIS for persistent symptoms had no recurrences. The re-intervention rate across the study period was similar between the groups (*p* = 0.888). Only 5 (8.8%) patients required escalation of treatment to LIS across the study period (Fig. 2).

### Incontinence

Faecal incontinence was reported on initial follow-up in 8.1% of group 1 and 10% of group 2 patients (*p* = 0.793). Long-term incontinence was assessed using the Pescatori score on follow-up survey, with a mean score of 0 ± 0.0 vs. 0.8 ± 1.6 in groups 1 and 2 respectively. This represents a significantly higher long-term incontinence rate in group 2 (0% vs. 10%, *p* = 0.010). The two patients with persistent incontinence were male and reported flatus-only incontinence, on a daily or weekly basis (Pescatori score A2 and A3). One patient, aged 52, had a history of recurrent anal fissures, for which he had reported a history of BT use previously. On follow-up survey he reported recurrence of fissure symptoms but has not required further surgical intervention. The second patient, a 71-year-old, has otherwise had an unremarkable recovery and reports no recurrence of fissure symptoms. Subgroup analysis based on gender shows a significant increased rate of long-term incontinence in men compared to women after BT treatment (*p* = 0.036). Of the five patients who required subsequent LIS for management of the CAF, two were female and no patient reported incontinence symptoms after the sphincter-dividing treatment.

### Overall satisfaction

There was a 65% response rate to the follow-up survey, with a median long-term follow-up of 34.3 (range 0.4–93) months. Overall treatment satisfaction was assessed using a Likert scale (1 = unsatisfied, 2 = neutral, 3 = satisfied, 4 = very satisfied), with a similar overall satisfaction rate of 3 out of 4 between the groups (*p* = 0.469).

## Discussion

Chronic anal fissure is a recurrent and persistent pathology which is challenging to manage. Lateral internal sphincterotomy has superior healing rate and low recurrence rate compared to topical treatment. However, it is associated with permanent incontinence in up to 10% of patients [7]. It is, therefore, logical to adopt a step-up approach starting with the least invasive treatment first.

Chemical sphincterotomy with BT has been found in systematic reviews to only be marginally superior to topical treatments [3, 20]. The addition of debridement of the fissure to BT injection is thought to aid healing by removing the chronic fibrosis whilst addressing the IAS spasm at the same time [15].

This retrospective study of 57 patients comparing combination treatment of BT and FIS versus BT only found no significant difference in the primary outcome of fissure healing (56.7% vs. 50%, *p* = 0.561). Additionally, the secondary outcomes of persistence rate, recurrence rate and re-intervention rate were similar between the two interventions. Of note, faecal incontinence was transient in the BT and FIS arm (group 1), whereas on long-term follow-up of a median of 34.3 months, persistent flatus incontinence was found in 10% of the BT-only arm (*p* = 0.01).

There are three comparative studies in the literature that examine the outcomes of CAF following BT with FIS versus BT alone with conflicting results [16–18]. Karabulut [17] reported no significant difference in complete healing rate between the two groups (77.8% vs. 61%, ns) which is consistent with the findings of this study. Similarly, in both studies there was a trend towards improved healing rate with BT and FIS despite failing to reach statistical significance. Nagle et al. [18], on the other hand, reported higher healing rate with BT and FIS compared to BT alone (47% vs. 17%). However, a healing rate of 17% with BT alone is very low compared to our reported healing rate and may account for their findings of superior outcome with BT and FIS. Winter [16] reported on recurrence rate rather than healing rate and showed fewer recurrences with BT and FIS compared to BT alone (31% vs. 56%). Our recurrence rates of 37.8% and 30% were relatively higher than those reported by Nagle [18] (22% vs. 23%) and Karabulut [17], who reported no recurrence in either group. This could be the result of differences in follow-up duration between the studies as median follow-up was 5 months in Karabulut [17], whereas median follow-up was 12 and 34 months in Nagle [18] and our study respectively. As expected, the recurrence rates reported increase in those studies with the increase in follow-up duration.

The ACPGBI guidelines recommend that persistent or recurrent CAF following an initial dose of BT should be considered for a subsequent dose [7]. However, BT injection has restricted funding in Australia and subsequent doses add a financial burden to the patient. We therefore found 5.4% of group 1 and 15% of group 2 patients progressed to LIS following persistent or recurrent symptoms. Similarly, Winter [16] found 5.8% and 34% required LIS, with significantly more BT-only patients requiring sphincter-dividing management (*p* < 0.001). Given more patients in the BT group required LIS, though not significant in our cohort, managing the chronic fibrotic wound with a fissurectomy may impact long-term outcomes, despite no significant difference in initial healing rate. LIS has a reported incontinence rate of 3.9–10%, and so treatment algorithms that can avoid a sphincterotomy are ideal. Therefore, there is a need to further investigate this trend of combination treatment resulting in fewer escalations to LIS.

There are several limitations with this study. Firstly as a retrospective review, there was missing data due to loss to follow-up. Furthermore, outcome results were dependent on interpretation of clinic notes. Additionally, with fissure healing taking several weeks, the definition of ‘healing’ included those with ongoing but improved symptoms as initial review ranged between 1.1 and 15.9 weeks post-procedure. Our results are limited by a small sample size and as expected due to the known inferior outcomes of the sphincter-sparing treatment, 15% of patients required further surgical treatment after our initial intervention. Finally, there is a risk of recall bias when collecting data from the follow-up survey. Despite this, our study provides long-term outcomes with sphincter-sparing treatment of chronic anal fissure.

## Conclusion

Botulinum toxin and fissurectomy does not improve the healing rate of CAF compared to botulinum toxin alone. Therefore, we recommend botulinum toxin injection alone as a second-line treatment of CAF in patients who fail topical treatment. Further prospective, randomised studies are needed to strengthen evidence for the use of combination treatment.

## Data Availability

Raw data is not publicly available to preserve individual’s privacy and in accordance with the ethics application approved by the Nepean Blue Mountains Local Health District Human Research Ethics Committee.
